# Associations between Physical Activity Level and Health Services Use in Spanish Adults

**DOI:** 10.3390/ijerph19148867

**Published:** 2022-07-21

**Authors:** Ángel Denche-Zamorano, María Mendoza-Muñoz, Jorge Carlos-Vivas, Laura Muñoz-Bermejo, Jorge Rojo-Ramos, Frano Giakoni-Ramírez, Andrés Godoy-Cumillaf, Sabina Barrios-Fernandez

**Affiliations:** 1 Health Economy Motricity and Education (HEME), Faculty of Sport Sciences, University of Extremadura, 10003 Caceres, Spain; andeza04@alumnos.unex.es; 2 Research Group on Physical and Health Literacy and Health-Related Quality of Life (PHYQOL), Faculty of Sport Sciences, University of Extremadura, 10003 Caceres, Spain; 3 Departamento de Desporto e Saúde, Escola de Saúde e Desenvolvimento Humano, Universidade de Évora, 7004-516 Evora, Portugal; 4 Promoting a Healthy Society Research Group (PHeSO), Faculty of Sport Sciences, University of Extremadura, 10003 Caceres, Spain; 5 Social Impact and Innovation in Health (InHEALTH), University of Extremadura, 10003 Caceres, Spain; lauramunoz@unex.es (L.M.-B.); jorgerr@unex.es (J.R.-R.); sabinabarrios@unex.es (S.B.-F.); 6 Faculty of Education and Social Sciences, Universidad Andres Bello, Las Condes 7550000, Chile; frano.giakoni@unab.cl; 7 Grupo de Investigación en Educación Física, Salud y Calidad de Vida, Facultad de Educación, Universidad Autónoma de Chile, Temuco 4810101, Chile; andres.godoy@uautonoma.cl

**Keywords:** physical activity, physical activity level, sedentariness, health services, health costs

## Abstract

One of the main concerns of governments and organisations worldwide is the cost and burden of health services, with sedentary lifestyles being a significant impacting factor, and physical activity is one of the potential solutions. Therefore, this study aims to analyse the possible associations between the physical activity level, hospitalisation prevalence, and the use and number of visits to emergency services in the Spanish population, examining potential differences between sex and age groups. This is a cross-sectional study based on data from the Spanish National Health Survey 2017 (ENSE 2017), the last one before the COVID-19 pandemic, with 17,199 participants. A descriptive analysis was performed using median and interquartile range (continuous variables) and absolute and relative frequencies (ordinal variables). Intergroup differences were analysed with non-parametric tests: chi-square and z-test for independent proportions (categorical variables), and Kruskal–Wallis and Mann–Whitney U (continuous variables). Spearman’s rho was used to study correlations between variables. A multiple binary regression analysis was performed to predict hospitalisations. Hospitalisations and emergency services use showed a dependence relation with the physical activity level (*p* < 0.001): those who performed moderate and/or vigorous physical activity used those services less than sedentary individuals and those whose only activity was walking. Thus, associations could be drawn between the hospitalisation prevalence, the use and number of visits to emergency departments, and the physical activity level in the Spanish population aged 18–69 years in the pre-pandemic period.

## 1. Introduction

In recent decades, the burdens and costs of public health services have been increasing, and they are expected to rise further [1,2] as a result of several health determinants, such as population ageing [3,4], sedentary habits [5,6] and an expected increase in the prevalence of non-communicable diseases [7,8]. Additionally, the effects of the COVID-19 pandemic [9,10] must be added to this equation. Healthcare costs are one of the main concerns of governments and organisations worldwide and present one of the most significant challenges to sustainability for health policymakers, managers, and researchers [11]. In Spain, health expenditure represents around 9% of the Gross Domestic Product (GDP), 1% below the European average; in 2017, healthcare costs represented an average of 2371 € per citizen, compared to 2884 € in Europe [12]. This contrasts with the United States, where health expenditure rises to 16% of GDP [13]. Part of this expenditure is due to hospitalisations and the use of emergency services; in Spain, there are four million hospitalisations per year, with an average cost of 4746 euros per hospitalisation, and the use of emergency services represents a cost of 31 million euros [14].

Physical activity is defined as any bodily movement produced by skeletal muscles that require energy expenditure [15] and is related to different health benefits: better weight control [16,17], glycemic control [18,19], pain management [20,21], and psychiatric symptomatology [21,22,23,24], as well as lower risk of certain types of cancer [25], among others. It is therefore conclusive that exercise promotes better health-related quality of life [26,27]. The World Health Organization (WHO) Guidelines on Physical Activity and Sedentary Behavior recommend performing 150–300 min of moderate physical activity per week, 75–150 min at a vigorous intensity, or a combination of both [28]. However, according to the Spanish General Secretary for Health Information [29], more than one-third (35.3%) of the population aged 15–69 years do not achieve these recommendations. Non-compliance with these recommendations is more frequent in women (37%) than in men (33.5%), and less physical activity is performed as age increases. Thus, it is reported that among those 15–24 years old, almost 34% engage in vigorous physical activity, while this percentage drops to 17.5% among those 55–69 years old. Moreover, among those 15–24 years old, almost 28% present a low physical activity level, a proportion that rises to nearly 38% among those 55–69 years old, increasing the risk of potential adverse health consequences [30,31]. As the WHO states, sedentary behaviours are defined as any waking behaviour characterised by an energy expenditure of 1.5 METs or less while sitting, reclining, or lying down, while physical inactivity is understood as performing less physical activity level than recommended. This can lead to adverse health consequences and increase the use of health services [32,33,34]. Therefore, increasing physical activity could generate substantial economic gains for the global economy [35].

An active lifestyle throughout life is a protective factor for some older peoples’ more prevalent health problems. Physical activity is one of the active ageing components. Some positive effects include reducing the impact of chronic and mental illnesses, pain, falls, fractures, and mortality risk factors [36], which could help reduce the costs of health service use [37,38]. By contrast, a sedentary lifestyle [39] and physical inactivity are associated with premature and pathological ageing, and with a variety of chronic conditions [40], which may result in increased demand for health services [41]. Thus, establishing physical activity programmes for adolescents, adults, and the elderly could lead to lower health service use, reducing health expenditure [42,43,44].

A study found that the COVID-19 lockdown resulted in decreased physical activity levels, with men (sex), individuals with primary and secondary studies (educational level), and the unemployed (employment status) showing the highest reductions [45]. Moreover, there was a significant decrease in moderate physical activity in Spanish adults with chronic conditions and a significant decline in vigorous physical activity in men with chronic diseases and multimorbidity [46]. Furthermore, the COVID-19 pandemic has influenced the Spanish adult population’s eating and sleeping habits, physical activity, and sedentary behaviour [45,46,47].

Therefore, this research aimed to study the associations between the physical activity level and the hospitalisation prevalence and the number of visits to the emergency services in members of the Spanish population between 18 and 69 years of age, considering sex and age group.

## 2. Materials and Methods

### 2.1. Desing and Participants

A descriptive correlational study was conducted using data obtained from the Adult Questionnaire from the ENSE 2017 [47]. This survey is developed by the Ministry of Health, Consumption and Social Welfare and the National Institute of Statistics every five years. Their objectives include identifying health-related factors that enable the planning, evaluation, and readjustment of health policies to make them as effective and efficient as possible. The last ENSE was conducted in 2017, before the COVID-2019 pandemic. The results of the survey will allow for the establishment of a comparative framework, with the following survey expected to be completed in 2023, in a post-pandemic context.

**Figure 1:**
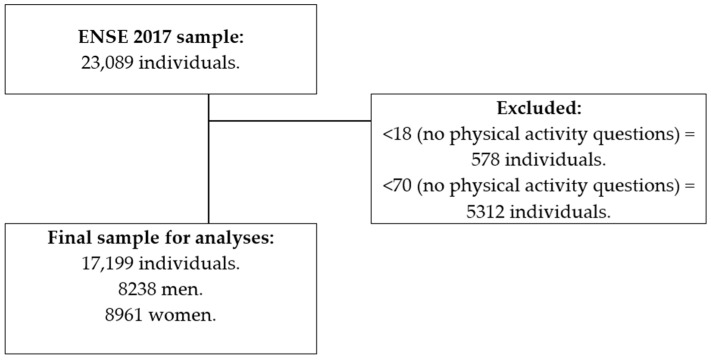
Chart outlining the study sample’s eligibility criteria.

The ENSE 2017 interviewed 23,089 individuals using a random stratified three-phase sampling system [48], including 10,595 men and 12,494 women over 15 years of age, residing in Spain. The inclusion criterion included being between 18 and 69 years. Participants under 18 (578 individuals) and over 70 (5312 individuals) were excluded, as they were not questioned about their physical activity level. Finally, the sample for this research was composed of 17,199 individuals (8238 men and 8961 women) as shown in Figure 1.

### 2.2. Measures and Variables

Below are the ENSE 17 sections where data were collected and the ad hoc variables constructed for this study.

Age: the numerical value was extracted from the ENSE 2017 to characterise the sample and create the age groups variable, which included young people (18–34 years), young adults (35–49 years), older adults (50–64 years) and older (65–69 years).

Sex: males and females were considered in the ENSE 17; they were used in this study to characterise the sample and construct subgroups by sex.

Physical Activity Index (PAI): created with the answers to the Spanish version of the International Physical Activity Questionnaire (IPAQ) [49] and computed by applying factors to the responses provided to items P. 113, P. 114, P. 115, and P. 116; and whose formula and factors have been used in previous research [50]. For the analyses that included this variable, 58 participants were excluded because they answered “don’t know”, or did not answer (NS/NC) to some items p. 113–117.

Physical Activity Level: derived from the PAI. It consists of six levels, two levels with people who had a PAI = 0, and four levels with people who had a PAI > 0. People with PAI = 0 were grouped into two levels, according to the answers given to item Q. 117 (“now think about how much time you spent walking in the last seven days”), discriminating between physically inactive (did not walk) and walkers (those whose only physical activity was walking). The six physical activity levels were: Inactive (PAI = 0; to the ENSE 17 question Q. 117 (“now think about how much time you spent walking in the last seven days”, they answered “no day more than ten consecutive minutes”)); Walkers (PAI = 0; to Q. 117, they answered that they walked at least one day a week, more than ten consecutive minutes); Low (PAI = 1–15, representing the population 75th percentile); Medium (PAI = 16–30, 90th percentile); High (PAI between 31–45, 95th percentile); and Very high (PAI > 45, above the 95th percentile).

Hospitalisation prevalence: data were extracted from question 66, “during the last 12 months, did you need to be admitted as a patient for at least one night excluding childbirth or caesarean section?” The available response options were “yes” or “no”.

Use of emergency services: data were extracted from question 78, “during the last 12 months, have you had to use any emergency services for any problem or illness?” The available response options were “yes” or “no”.

Number of visits to emergency services: data were obtained from question 79, “and in total, how many times did you have to use an emergency service in the last 12 months?” with the response options being the total number of visits or “don’t know/no answer”. Ten participants were excluded from the analyses with this variable because they answered “NS/NC”, and two participants were excluded because they had visited emergency services 50 and 200 times, respectively, being extreme values.

### 2.3. Ethical Concerns

According to Regulation 2016/679 of the European Parliament and of the Council of 27 April 2016 on the protection of individuals about the processing of personal data and the free movement of personal data and derogating from Directive 95/46/EC [51], anonymous files for public use are not considered confidential. Therefore, approval by a bioethics committee is not required.

### 2.4. Statistical Analysis

Statistical analyses were carried out with the Statistical Package for the Social Sciences software version 25 (IBM SPSS, Armonk, NY, USA). A descriptive analysis was performed using the median and the interquartile range, with mean and standard deviation (continuous variables) and absolute and relative frequencies (ordinal variables). A Chi-Square test was performed to analyse the dependence relationships between the PAL and the rest of the categorical variables of interest. In these, a pairwise z-test of independent proportions was carried out to analyse potential differences in inter-group proportions (ordinal variables). The Kruskal–Wallis’s and Mann–Whitney’s U-test (continuous variables) were employed to explore the dependence between variables and intergroup differences, respectively. Spearman’s rho was used to analyse the correlations between variables. A multiple binary logistic binary model was used to study the effects of predictor variables (age, PAL, and use of emergency services) on hospitalisations. Two-sided *p*-values ≤ 0.05 were considered statistically significant.

## 3. Results

Table 1 presents the sociodemographic characteristics of the sample; it includes information on the hospitalisation prevalence, use and number of visits to emergency services within the last year, and the physical activity level in the Spanish population aged 18–69 years, according to the ENSE 2017. Concerning sex differences, women reported a higher number of hospitalisations, higher use of emergency services, and lower physical activity levels than men.

Table 2 shows the dependency ratios of hospitalisation prevalence according to age groups, both in the total population and by sex. As seen, the hospitalisation proportions (at least once in the 12 months) increase with age.

In the same line, dependency ratios were found between the hospitalisation prevalence and the physical activity level in the general population and the two age groups (Table 3), with more hospitalisations as the physical activity levels decreased, and higher use in women compared to men.

Table 4 reports the associations between the hospitalization prevalence and the physical activity level by age group, including dependency between these variables. Those who only did walking (“walkers”) showed lower hospitalization proportions than those in the “Inactive” in all cases.

Table 5 shows the associations between the use of emergency services in the 12 months preceding the survey and the physical activity level by age group, including dependency between these variables. In all the age groups, women used the emergency services more than men.

In Table 6, associations between the use of emergency services and the physical activity level in the Spanish population by sex can be found. Dependency relationships were found between both variables in the general population as well as in the men’s and women’s groups. Women with lower physical activity levels used emergency services more, while those with higher levels used them less than their male counterparts.

Table 7 displays the Associations between the use of emergency services and physical activity level. Dependence relationships were found between the prevalence of use of emergency services and physical activity level in every age group.

Table 8 shows the association between the number of visits to the emergency services according to physical activity level and sex. Women visited emergency services more than men in all cases, but the use of emergency services decreased as the physical activity level increased both in men and women.

The analysis of correlations revealed weak correlations between the physical activity level and (1) the hospitalisation prevalence (rho: −0.086. *p* < 0.001), (2) the use of emergency services (rho = −0.085. *p* < 0.001) and (3) the number of visits emergency services (rho = −0.091. *p* < 0.001).

Finally, after performing a binary logistic regression analysis about hospitalisations as shown in Table 9. Those who were younger, women, people who had not used emergency services, and those with active lifestyles showed a lower risk of being hospitalised. Thus, the logistic regression model explained 14.1% (Nagelkerke R^2^) of the variance in hospitalization.

## 4. Discussion

### 4.1. Main Findings and Theoretical Implications

The main purpose of this research was to examine the associations between the physical activity level and the use of health services by those between 18 and 69 years of age in the Spanish population during the last pre-pandemic period using the ENSE 2017 data [52]. The main contributions were the discovery of associations in the general population, in both sexes and the different age groups, between the different variables. A dependency ratio was found between the physical activity level and (1) the hospitalization prevalence and (2) the use of emergency services, discovering that belonging to the “Inactive” group was related to a higher hospitalisation prevalence and use of emergency services compared with those who only did walking (“Walkers”).

Regarding the hospitalisation prevalence, no differences between the sexes were found, although there was an increase as age groups increased. In the general population, the hospitalisation prevalence ranged from 4.6% in young people to 12.2% in older people. In men, the percentage points increased more than three times between younger and older age groups (4.1% vs. 13.5%), a difference of 11.4 percentage points. In women, the differences were more than twice as large between younger and older (5.0% vs. 11.1%). Likewise, dependency ratios were found between the hospitalisation prevalence and physical activity level in the general population and both sexes. In the inactive group, the hospitalisation prevalence was 11.6%, 7.8 percentage points higher than the “High” and “Very high” groups, with similar rates in both sexes. Between “Inactive” and “Walkers”, a 4-percentage point difference in favour of “Walkers” was found, being 3.5 points in men and 4.3 points in women. Daily activity in adults and older adults predicts fewer future hospitalisations, reducing the length of hospital stays and the number of admissions in a previous study [53].

Moreover, dependency ratios were also found in the general population and the sex groups in emergency services use. In the general population, young people had the highest prevalence of emergency services use (35.0%), with older people (24.7%) reporting the lowest use. Prevalence in young men was 30.9%, versus 23.9% in adults and 24.7% in older adults. In the women’s group, the prevalence was 38.6 in younger women, 27.9 in adults, and 29.0% in older women, with differences of 7.7, 4, and 4.3 percentage points more than men of the same age. Dependency ratios were also found between the use of emergency services and the physical activity level in the general population, as well as in the men and women groups, with a 16.4 percentage point difference between the “Inactive” and “Very high” levels, a 14.6-point difference in men and a 16.1-point difference in women. Between those categorised as “Inactive” and “Walkers”, a 9.6 percentage-point difference was found in prevalence in the general population, 8.7 in men and 10.6 in women. These associations were confirmed in all age groups, decreasing as the physical activity levels increased. In the elderly, the prevalence of emergency services use dropped from 41.1% in the “Inactive” to 15.4% in the “High” physical activity level group. Differences of 15–20 percentage points were reported between the “Inactive” and the “Very high” physical activity level groups in young, adults, and older people. Usage differences between the “Inactive” and “Walkers” groups widened as age increased, from 5.1 percentage points in young people to 12.1 in adults and 13.8 in older individuals. Concerning the number of visits to emergency departments in the 12 months before the 2017 ENSE, significant differences were found between “Inactive” and “Walkers”, and between these and the rest of the groups, in the general population and the sex groups. The mean number of visits in men went from 0.66 in “Inactive” to 0.28 in the “Very high” physical activity level. In women, the mean number of visits was 0.99 in “Inactive” compared to 0.44 in “Medium” physical activity level. A US study found that adults who engaged in regular physical activity used more preventive and consultative services and significantly fewer inpatient, emergency, home health care, and prescription drug services [38]. Some studies suggest that sex is linked to the use of health services, being that women use them more often [54,55,56].

Thus, 28.9% of the Spanish population used emergency services in the 12 months before the ENSE 2017, representing a 26.5% prevalence in men and 31.1% in women. Possible explanations for this difference are multifactorial: women often experience a poorer self-perception of their health status, so they show more significant concern about suspicious symptomatology [57]; gender-related differences [58]; willingness to receive and follow medical advice [59]; they present higher prevalence and degree of pain due to hormonal, biological and contextual causes [60]; and poorer mental health [61], among others. On the other hand, age is also related to the higher use of health services, including outpatient and inpatient care for chronic diseases, due, among other reasons, to sedentary lifestyles [62]. Other studies have found associations between age and higher use of medicines, laboratory examinations, and visits to health centres, hospitals, and emergency services [63,64].

### 4.2. Practical Implications

The importance of this study relies on the analyses of the associations between the physical activity level and the use of health services in the Spanish population during the last period before the COVID-19 pandemic, which could serve as a frame of reference for future research examining post-pandemic periods, as the ENSE is addressed every 5 years. Furthermore, by the negative consequences caused by the pandemic on the daily habits of the Spanish population concerning physical activity and sedentary behaviour, this study can serve as a framework to study the post-pandemic situation and verify whether the use of medical services by the Spanish population increases [45,46,65].

Given these data, the associations found showed that the inactive population generally uses more health services compared with those who at least walked. Additionally, those who only walked used more health services than those who were more active. Moreover, the results indicated that the number of visits to emergency services was higher in the inactive group than in the groups who walked or performed moderate and/or vigorous physical activity. Although various research recommends performing medium to high physical activity to improve their health benefits [37,38], our study design does not allow us to establish cause-effect relations.

### 4.3. Limitations and Future Lines

The most important limitation is that this type of study design does not allow us to establish cause-effect relations, thus future lines should include longitudinal studies; therefore, it would be recommended that the survey should retain all the analysed variables. Another limitation is that the differences between moderate and vigorous physical activity couldn’t be analysed, which should be considered in future research. Another limitation is the lack of participants’ medical histories, physical activity objectives, and physiological data, including follow-ups; a methodological improvement that could be implemented could be to perform a 24-h compositional analysis, including devices to quantify physical activity intensity or other measures to overcome some of the limitations of survey-based studies.

## 5. Conclusions

This study found associations between the hospitalisation prevalence, the use and number of visits to emergency departments, and the physical activity level in the Spanish population aged 18–69 years in the pre-pandemic period, analysed by sex and age groups.

These results need to be confirmed with longitudinal studies in order to recommend the PA programs or “sports prescription” implementation as a cost-effective alternative to reduce health expenditure.

## Figures and Tables

**Table 1:** Sociodemographic characteristics of the sample.

Age (Years)	Men = 8238	Women = 8961	Total = 17,199	*p*
Median (IQR)	47 (20)	47 (21)	47 (21)	0.467
Mean (SD)	46.7 (13.2)	46.9 (13.3)	46.8 (13.2)	-
Age Group (years)	Men *n* (%)	Women *n* (%)	Total *n* (%)	*p* *
18–34	1573 (19.1) _a_	1743 (19.5) _a_	3316 (19.3)	0.227
35–49	3007 (36.5) _a_	3188 (35.6) _a_	6195 (36.0)	
50–64	2874 (34.9) _a_	3103 (34.6) _a_	5977 (34.8)	
65–69	784 (9.5) _a_	927 (10.3) _a_	1711 (9.9)	
Hospitalization	Men = 8238 *n* (%)	Women = 8961 *n* (%)	Total = 17,199 *n* (%)	*p* *
Yes	601 (7.3) _a_	615 (6.9) _a_	1216 (7.1)	0.269
No	7637 (92.7) _a_	8346 (93.1) _a_	15,983 (92.9)	
Use of Emergency Services	Men = 8238 *n* (%)	Women = 8961 *n* (%)	Total = 17,199 *n* (%)	*p* *
Yes	2181 (26.5) _a_	2784 (31.1) _b_	4965 (28.9)	<0.001
No	6057 (73.5) _a_	6177 (68.9) _b_	12,234 (71.1)	
Visits to Emergency Services	Men = 8234	Women = 8953	Total = 17,187	*p*
Median (IQR)	0 (1)	0 (1)	0 (1)	<0.001
Mean (SD)	0.42 (1.02)	0.60 (1.44)	0.51 (1.27)	-
CI (95%)	0.40–0.44	0.57–0.63	0.49–0.53	-
PAL	Men = 8199 *n* (%)	Women = 8942 *n* (%)	Total = 17,141 *n* (%)	*p* *
Inactive (PAI = 0)	1156 (14.1)	1326 (14.8)	2482 (14.5)	<0.001
Walkers (PAI = 0)	3335 (40.7)	4566 (51.1)	7901 (46.1)	
Low (PAI = 1–15)	1077 (13.1)	1262 (14.1)	2339 (13.6)	
Medium PAI = 16–30)	1273 (15.5)	1076 (12.0)	2349 (13.7)	
High (PAI = 31–45)	877 (10.7)	476 (5.3)	1353 (7.9)	
Very High (PAI > 45)	481 (5.9)	236 (2.6)	717 (4.2)	

IQR: interquartile range; SD: standard deviation *n*: participants; %: percentage; PAL: Physical Activity Level, PAI: Physical Activity Index; only considers vigorous and moderate physical activity. Scores between 0 and 67.5; Inactive: PAI = 0; reports not walking at least one day a week for more than 10 min at a time; Walkers PAI = 0; reports walking at least one day a week for more than 10 min at a time; Low: PAI = 1–15; Medium: PAI = 16–30; High: PAI = 31–45; Very high: PAI > 45; *p*: *p*-value from Mann–Whitney U test; *p* *: *p*-value from Chi-square test; _a_, _b_: Pairwise z-test for independent proportions, each subscript represents a subset, whose column proportions differ significantly at the 0.05 level; CI: Confidence interval at 0.05 level of significance.

**Table 2:** Age group relations and the hospitalisation prevalence in the general Spanish population aged 18–69 years, compared by sex, according to the ENSE 2017.

	Men (*n* = 8238)			Women (*n* = 8961)			Total (*n* = 17,199)		
Age Group (Years)	Yes	No	*p*	Yes	No	*p*	Yes	No	*p*
18–34 (*n* = 3316)	64 (4.1) _a_	1509 (95.9) _b_	<0.001	87 (5.0) _a_	1656 (95.0) _b_	<0.001	151 (4.6) _a_	3165 (95.4) _b_	<0.001
35–49 (*n* = 6195)	170 (5.7) _a_	2837 (94.3) _b_		176 (5.5) _a_	3012 (94.5) _b_		346 (5.6) _a_	5849 (94.4) _b_	
50–64 (*n* = 5977)	261 (9.1) _a_	2613 (90.9) _b_		249 (8.0) _a_	2854 (92.0) _b_		510 (8.5) _a_	5467 (91.5) _b_	
65–69 (*n* = 1711)	106 (13.5) _a_	678 (86.5) _b_		103 (11.1) _a_	824 (88.9) _b_		209 (12.2) _a_	1502 (87.8) _b_	
Total (*n* = 17,199)	601 (7.3)	7637 (92.7)		615 (6.9)	8346 (93.1)		1216 (7.1)	15,983 (92.9)	

Values presented in absolute and relative frequencies; *n*: number of participants; Yes: hospitalised at least once in the last 12 months; No: not hospitalised in the previous 12 months; *p*: *p*-value from chi-square test; _a_, _b_: Pairwise z-test for independent proportions. Each subscript corresponds to a subset whose column proportions do not differ from each other at the 0.05 level.

**Table 3:** Associations between the hospitalisation prevalence and physical activity level in the general Spanish population aged 18–69 years compared by sex, according to the ENSE 2017.

	Men (*n* = 8199)			Women (*n* = 8942)			Total (*n* = 17,141)		
PAL	Yes	No	*p*	Yes	No	*p*	Yes	No	*p*
Inactive *n* = 2482	137 (11.9) _a_	1019 (88.1) _b_	0.001	150 (11.3) _a_	1176 (88.7) _b_	<0.001	287 (11.6) _a_	2195 (88.4) _b_	<0.001
Walkers *n* = 7901	281 (8.4) _a_	3054 (91.6) _b_		320 (7.0) _a_	4246 (93.0) _a_		601 (7.6) _a_	7300 (92.4) _b_	
Low *n* = 2339	58 (5.4) _a_	1019 (94.6) _b_		67 (5.3) _a_	1195 (94.7) _b_		125 (5.3) _a_	2214 (94.7) _b_	
Medium *n* = 2349	73 (5.7) _a_	1200 (94.3) _b_		50 (4.6) _a_	1026 (95.4) _b_		123 (5.2) _a_	2226 (94.8) _b_	
High *n* = 1353	32 (3.6) _a_	845 (96.4) _b_		19 (4.0) _a_	457 (96.0) _b_		51 (3.8) _a_	1302 (96.2) _b_	
Very High *n* = 717	18 (3.7) _a_	463 (96.3) _b_		9 (3.8) _a_	227 (96.2) _b_		27 (3.8) _a_	690 (96.2) _b_	
Total *n* = 17,141	599 (7.3)	7600 (92.7)		615 (6.9)	8327 (93.1)		1214 (7.1)	15,927 (92.9)	

Values presented in absolute and relative frequencies; *n*: number of participants; PAL (Physical Activity Level) PAI: Physical Activity Index; only considers vigorous and moderate physical activity. Scores between 0 and 67.5; Inactive: PAI = 0; reports not walking at least one day a week for more than 10 min at a time; Walkers PAI = 0; reports walking at least one day a week for more than 10 min at a time; Low: PAI = 1–15; Medium: PAI = 16–30; High: PAI = 31–45; Very high: PAI > 45; Yes: hospitalised at least once in the last 12 months; No: not hospitalised in the last 12 months; *p*: *p*-value from chi-square test; _a_, _b_: Pairwise z-test for independent proportions. Each subscript corresponds to a subset whose column proportions do not differ from each other at the 0.05 level.

**Table 4:** Associations between the hospitalisation prevalence and physical activity level in the general Spanish population aged 18–69 years compared by age, according to the ENSE 2017.

	18–34 Years (*n* = 3297)			35–49 Years (*n* = 6177)			50–64 Years (*n* = 5956)			65–69 Years (*n* = 1711)			Total (*n* = 17,141)		
PAL	Yes *n* (%)	No *n* (%)	*p*	Yes *n* (%)	No *n* (%)	*p*	Yes *n* (%)	No *n* (%)	*p*	Yes *n* (%)	No *n* (%)	*p*	Yes *n* (%)	No *n* (%)	*p*
Inactive *n* = 2485	33 (7.8) _a_	388 (92.2) _b_	0.002	79 (8.4) _a_	856 (91.6) _b_	<0.001	127 (14.5) _a_	751 (85.5) _b_	<0.001	48 (19.4) _a_	200 (80.6) _b_	0.002	287 (11.6) _a_	2195 (88.4) _b_	<0.001
Walkers *n* = 7910	61 (5.0) _a_	1158 (95.0) _a_		158 (6.2) _a_	2407 (93.8) _a_		263 (8.4) _a_	2861 (91.6) _a_		119 (12.0) _a_	874 (88.0) _a_		601 (7.6) _a_	7300 (92.4) _b_	
Low *n* = 2342	22 (4.6) _a_	456 (95.4) _a_		38 (3.9) _a_	932 (96.1) _b_		52 (7.5) _a_	644 (92.5) _a_		13 (6.7) _a_	182 (93.3) _b_		125 (5.3) _a_	2214 (94.7) _b_	
Medium *n* = 2353	18 (3.4) _a_	512 (96.6) _a_		43 (4.9) _a_	838 (95.1) _a_		39 (5.2) _a_	704 (94.8) _b_		23 (11.8) _a_	172 (88.2) _a_		123 (5.2) _a_	2226 (94.8) _b_	
High *n* = 1355	14 (3.2) _a_	427 (96.8) _a_		21 (3.8) _a_	533 (96.2) _a_		12 (3.9) _a_	294 (96.1) _b_		4 (7.7) _a_	48 (92.3) _a_		51 (3.8) _a_	1302 (96.2) _b_	
Very High *n* = 734	3 (1.4) _a_	205 (98.6) _b_		7 (2.6) _a_	265 (97.4) _b_		15 (7.2) _a_	194 (92.8) _a_		2 (7.1) _a_	26 (92.9) _a_		27 (3.8) _a_	690 (96.2) _b_	
Total *n* = 17,141	151 (4.6)	3146 (95.4)		346 (5.6)	5831 (94.4)		508 (8.5)	5448 (91.5)		209 (12.2)	1502 (87.8)		1214 (7.1)	15,927 (92.9)	

Values presented in absolute and relative frequencies; *n*: number of participants; %: percentage; PAL (Physical Activity Level); PAI: Physical Activity Index; only consider vigorous and moderate physical activity. Scores between 0 and 67.5; Inactive: PAI = 0; reports not walking at least one day a week for more than 10 min at a time; Walkers PAI = 0; reports walking at least one day a week for more than 10 min at a time; Low: PAI = 1–15; Medium: PAI = 16–30; High: PAI = 31–45; Very high: PAI > 45; Yes: hospitalised at least once in the last 12 months; No: not hospitalised in the last 12 months; *p*: *p*-value from chi-square test; _a_, _b_: Pairwise z-test for independent proportions. Each subscript corresponds to a subset whose column proportions do not differ from each other at the 0.05 level.

**Table 5:** Associations between the use of emergency services in the general Spanish population aged 18–69 years, compared by age, according to the ENSE 2017.

	Men (*n* = 8238)			Women (*n* = 8961)			Total (*n* = 17,199)		
Age Groups	Yes *n* (%)	No *n* (%)	*p*	Yes *n* (%)	No *n* (%)	*p*	Yes *n* (%)	No *n* (%)	*p*
18–34 years (*n* = 3316)	486 (30.9) _a_	1087 (69.1) _b_	<0.001	673 (38.6) _a_	1070 (61.4) _b_	<0.001	1159 (35.0) _a_	2157 (65.0) _b_	<0.001
35–49 years (*n* = 6195)	813 (27.0) _a_	2194 (73.0) _a_		977 (30.6) _a_	2211 (69.4) _a_		1790 (28.9) _a_	4405 (71.1) _a_	
50–64 years (*n* = 5977)	688 (23.9) _a_	2186 (76.1) _b_		865 (27.9) _a_	2238 (72.1) _b_		1553 (26.0) _a_	4424 (74.0) _b_	
65–69 years (*n* = 1711)	194 (24.7) _a_	590 (75.3) _a_		269 (29.0) _a_	658 (71.0) _a_		463 (27.1) _a_	1248 (72.9) _a_	
Total (*n* = 17,199)	2181 (26.5)	6057 (73.5)		2784 (31.1)	6177 (68.9)		4965 (28.8)	12,234 (71.2)	

Values presented in absolute and relative frequencies; *n*: number of participants; %: Percentage; Yes: used emergency services at least once in the last 12 months; No: did not use emergency services at least once in the last 12 months; *p*: *p*-value from chi-square test; _a_, _b_: Pairwise z-test for independent proportions. Each subscript corresponds to a subset whose column proportions do not differ from each other at the 0.05 level.

**Table 6:** Associations between the use of emergency services and physical activity level in the general Spanish population aged 18–69 years compared by sex, according to the ENSE 2017.

	Men (*n* = 8199)			Women (*n* = 8942)			Total (*n* = 17,141)		
PAL	Yes *n* (%)	No *n* (%)	*p*	Yes *n* (%)	No *n* (%)	*p*	Yes *n* (%)	No *n* (%)	*p*
Inactive *n* = 2482	407 (35.2) _a_	749 (64.8) _b_	0.002	550 (41.5) _a_	776 (58.5) _b_	<0.001	957 (38.6) _a_	1525 (61.4) _b_	<0.001
Walkers *n* = 7901	883 (26.5) _a_	2452 (73.5) _a_		1409 (30.9) _a_	3157 (69.1) _a_		2292 (29.0) _a_	5609 (71.0) _a_	
Low *n* = 2339	244 (22.7) _a_	833 (77.3) _b_		353 (28.0) _a_	909 (72.0) _b_		597 (25.5) _a_	1742 (74.5) _b_	
Medium *n* = 2349	315 (24.7) _a_	958 (75.3) _a_		285 (26.5) _a_	791 (73.5) _b_		600 (25.5) _a_	1749 (74.5) _b_	
High *n* = 1353	227 (25.9) _a_	650 (74.1) _a_		127 (26.7) _a_	349 (73.3) _b_		354 (26.2) _a_	999 (73.8) _b_	
Very High *n* = 717	99 (20.6) _a_	382 (79.4) _b_		60 (25.4) _a_	176 (74.6) _a_		159 (22.2) _a_	558 (77.8) _b_	
Total *n* = 17,141	2175 (26.5)	6024 (73.5)		2784 (31.1)	6158 (68.9)		4959 (28.9)	12,182 (71.1)	

Values presented in absolute and relative frequencies; *n*: number of participants; %: Percentage; PAL: Physical Activity Level); PAI: Physical Activity Index; only considers vigorous and moderate physical activity. Scores between 0 and 67.5; Inactive: PAI = 0; reports not walking at least one day a week for more than 10 min at a time; Walkers PAI = 0; reports walking at least one day a week for more than 10 min at a time; Low: PAI = 1–15; Medium: PAI = 16–30; High: PAI = 31–45; Very high: PAI > 45; Yes: used emergency services at least once in the last 12 months; No: did not use emergency services at least once in the last 12 months; *p*: *p*-value from chi-square test; _a_, _b_: Pairwise z-test for independent proportions. Each subscript corresponds to a subset whose column proportions do not differ from each other at the 0.05 level.

**Table 7:** Associations between the use of emergency services and physical activity level in the general Spanish population aged 18–69 years compared by age, according to the ENSE 2017.

	18–34 Years (*n* = 3297)			35–49 Years (*n* = 6177)			50–64 Years (*n* = 5956)			65–69 Years (*n* = 1711)			Total (*n* = 17,141)		
PAL	Yes *n* (%)	No *n* (%)	*p*	Yes *n* (%)	No *n* (%)	*p*	Yes *n* (%)	No *n* (%)	*p*	Yes *n* (%)	No *n* (%)	*p*	Yes *n* (%)	No *n* (%)	*p*
Inactive *n* = 2482	178 (42.3) _a_	243 (57.7) _b_	0.001	343 (36.7) _a_	592 (63.3) _b_	<0.001	334 (38.0) _a_	544 (62.0) _b_	<0.001	102 (41.1) _a_	146 (58.9) _b_	0.001	957 (38.6) _a_	1525 (61.4) _b_	<0.001
Walkers *n* = 7901	454 (37.2) _a_	765 (62.8) _b_		758 (29.6) _a_	1807 (70.4) _a_		809 (25.9) _a_	2315 (74.1) _a_		271 (27.3) _a_	722 (72.7) _a_		2292 (29.0) _a_	5609 (71.0) _a_	
Low *n* = 2339	149 (31.2) _a_	329 (68.8) _a_		265 (27.3) _a_	705 (72.7) _b_		139 (20.0) _a_	557 (80.0) _b_		44 (22.6) _a_	151 (77.4) _a_		597 (25.5) _a_	1742 (74.5) _b_	
Medium *n* = 2349	178 (33.6) _a_	352 (66.4) _a_		229 (26.0) _a_	652 (74.0) _b_		161 (21.7) _a_	582 (78.3) _b_		32 (16.4) _a_	163 (83.6) _b_		600 (25.5) _a_	1749 (74.5) _b_	
High *n* = 1353	144 (32.7) _a_	297 (67.3) _a_		134 (24.2) _a_	420 (75.8) _b_		68 (22.2) _a_	238 (77.8) _a_		8 (15.4) _a_	44 (84.6) _a_		354 (26.2) _a_	999 (73.8) _b_	
Very High *n* = 717	54 (26.0) _a_	154 (74.0) _b_		60 (22.1) _a_	212 (77.9) _b_		39 (18.7) _a_	170 (81.3) _b_		6 (21.4) _a_	22 (78.6) _a_		159 (22.2) _a_	558 (74.8) _b_	
Total *n* = 17,141	1157 (35.1)	2140 (64.9)		1789 (29.0)	4388 (71.0)		1550 (26.0)	4406 (74.0)		463 (27.1)	1248 (72.9)		4959 (28.9)	12,182 (71.1)	

Values presented in absolute and relative frequencies; *n*: number of participants; % Percentage; PAL (Physical Activity Level); PAI: Physical Activity Index; only considers vigorous and moderate physical activity. Scores between 0 and 67.5; Inactive: PAI = 0; reports not walking at least one day a week for more than 10 min at a time; Walkers PAI = 0; reports walking at least one day a week for more than 10 min at a time; Low: PAI = 1–15; Medium: PAI = 16–30; High: PAI = 31–45; Very high: PAI > 45; Yes: used emergency services at least once in the last 12 months; No: did not use emergency services at least once in the last 12 months; *p*: *p*-value from chi-square test; _a_, _b_: Pairwise z-test for independent proportions. Each subscript corresponds to a subset whose column proportions do not differ from each other at the 0.05 level.

**Table 8:** Associations between the number of visits to emergency services and physical activity level in the general Spanish population aged 18–69 years compared by sex, according to the ENSE 2017.

**Men**								
**PAL**	***n***	**Mean**	**(SD)**	**CI (95%)**	**Med**	**(IQR)**	***p***	*p* *
Inactive	1156	0.66	(1.39)	0.58–0.74	0	(1)	<0.001	_a_
Walkers	3333	0.43	(1.06)	0.39–0.46	0	(1)		_b_
Low	1076	0.34	(0.91)	0.29–0.40	0	(0)		_c_
Medium	1273	0.35	(0.83)	0.31–0.40	0	(0)		_bc_
High	877	0.37	(0.79)	0.32–0.42	0	(1)		_bc_
Very High	481	0.28	(0.65)	0.23–0.34	0	(0)		_c_
**Women**								
**PAL**	***n***	**Mean**	**(SD)**	**CI (95%)**	**Med**	**(IQR)**	***p***	*p* *
Inactive	1322	0.99	(2.22)	0.87–1.11	0	(1)	<0.001	_a_
Walkers	4562	0.58	(1.34)	0.54–0.61	0	(1)		_b_
Low	1262	0.46	(1.08)	0.40–0.52	0	(1)		_c_
Medium	1076	0.44	(1.09)	0.37–0.50	0	(1)		_c_
High	476	0.47	(1.04)	0.37–0.56	0	(1)		_b_
Very High	236	0.51	(1.30)	0.35–0.68	0	(1)		_b_
**Total**								
**PAL**	***n***	**Mean**	**(SD)**	**CI (95%)**	**Med**	**(IQR)**	***p***	*p* *
Inactive	2478	0.84	(1.89)	0.76–0.91	0	(1)	<0.001	_a_
Walkers	7895	0.51	(1.23)	0.49–0.54	0	(1)		_b_
Low	2338	0.41	(1.00)	0.37–0.45	0	(1)		_c_
Medium	2349	0.39	(0.96)	0.35–0.43	0	(1)		_c_
High	1353	0.41	(0.89)	0.36–0.45	0	(1)		_c_
Very High	717	0.36	(0.92)	0.29–0.43	0	(0)		_c_

PAL: Physical Activity Level; PAI: Physical Activity Index; only considers vigorous and moderate physical activity. Scores between 0 and 67.5; Inactive: PAI = 0; reports not walking at least one day a week for more than 10 min at a time; Walkers PAI = 0; reports walking at least one day a week for more than 10 min at a time; Low: PAI = 1–15; Medium: PAI = 16–30; High: PAI = 31–45; Very high: PAI > 45; *n*: participants; SD: standard deviation; CI: confidence interval; Med: Median; IQR: interquartile range; *p*: value from Kruskal-Wallis test; *p* *: *p*-value from Mann–Whitney U test; _a–c_: different subscripts denote significant intergroup differences at the 0.05 level using the Mann–Whitney U-test.

**Table 9:** Logarithmic binary regression model for the hospitalisation risk factor.

	B	SE	Wald	Df	Sig	Exp(B)	95% CI for EXP(B)	
							Lower	Upper
Years	0.031	0.003	154.816	1	<0.001	1.032	1.027	1.037
Inactive			56.134	5	<0.001			
Walkers	−0.350	0.079	19.473	1	<0.001	0.705	0.603	0.823
Low	−0.561	0.115	23.698	1	<0.001	0.571	0.455	0.715
Medium	−0.593	0.116	26.191	1	<0.001	0.553	0.440	0.694
High	−0.848	0.161	27.799	1	<0.001	0.428	0.313	0.587
Very High	−0.826	0.211	15.294	1	<0.001	0.438	0.289	0.662
Sex	−0.203	0.062	10.526	1	<0.001	0.817	0.723	0.923
Urgencies	−1.704	0.064	704.894	1	<0.001	0.182	0.160	0.206
Constant	−2.668	0.146	333.764	1	<0.001	0.069		

B: Understandarized beta; SE: Standard error of the regression; Wald: Wald Chi-Squared Test; Df: Degrees of freedom; Sig: Statistical significance; Exp: Exponential regression; CI: Confidence Interval. Inactive: PAI = 0; reports not walking at least one day a week for more than 10 min at a time; Walkers PAI = 0; reports walking at least one day a week for more than 10 min at a time; Low: PAI = 1–15; Medium: PAI = 16–30; High: PAI = 31–45; Very high: Sex: men or women; PAI > 45; Yes: used emergency services at least once in the last 12 months. Yes: used emergency services at least once in the last 12 months; No: did not use emergency services at least once in the last 12 months.

## Data Availability

Datasets will be available under reasonable request.
